# A four-step process for building sustainable access to diagnosis and treatment of Chagas disease

**DOI:** 10.26633/RPSP.2019.74

**Published:** 2019-09-20

**Authors:** Carolina Batista, Colin J Forsyth, Rafael Herazo, Marina Pereira Certo, Andrea Marchiol

**Affiliations:** Drugs for Neglected Diseases initiative, Chagas Treatment Access Project Drugs for Neglected Diseases initiative, Chagas Treatment Access Project Geneva Switzerland Drugs for Neglected Diseases initiative, Chagas Treatment Access Project, Geneva, Switzerland.

**Keywords:** Chagas disease, equity in access to health services, neglected diseases, drug development, Colombia, Enfermedad de Chagas, equidad en el acceso a los servicios de salud, enfermedades desatendidas, desarrollo de medicamentos, Colombia, Doença de Chagas, equidade no acesso aos serviços de saúde, doenças negligenciadas, desenvolvimento de medicamentos, Colômbia

## Abstract

The vast majority of people with Chagas disease (CD) are undiagnosed and untreated. Improving access to diagnosis and treatment for CD involves confronting a wide range of barriers. This report discusses a collaborative approach to eliminate barriers and increase the availability of CD testing and treatment. Potential areas for intervention are selected based on burden of disease, support of local champions, and commitment from national and local authorities. A 4D approach (diagnose, design, deliver, and demonstrate impact) is then implemented. The diagnose step involves gathering key stakeholders at a seminar to collaboratively identify important barriers and propose solutions. The design step creates a specific plan to act upon the seminar’s conclusions with consensus on core indicators. The deliver step entails implementing the plan at pilot locations, while simultaneously strengthening health system capacity for CD testing and treatment. Lastly, the demonstrate impact step compares baseline data with annual post-implementation data to measure progress. In Colombia, this approach has helped simplify testing procedures and increase CD testing and treatment access in pilot communities, though challenges remain. The 4D approach represents one of several pathways toward ensuring that the best therapeutic and diagnostic products reach people affected by neglected tropical diseases.

## RESUMEN

Una amplia mayoría de las personas con la enfermedad de Chagas no reciben diagnóstico ni tratamiento. Para mejorar el acceso al diagnóstico y al tratamiento de esta enfermedad es necesario superar multiples obstáculos. En el presente informe se considera un método colaborativo diseñado para superar las barreras y aumentar la disponibilidad de las pruebas de detección y el tratamiento de la enfermedad de Chagas. Se seleccionan los posibles ámbitos de intervención en función de la carga de enfermedad, el apoyo por parte de los líderes locales y el compromiso de las autoridades locales y nacionales. Posteriormente, se aplica un método 4D basado en cuatro pasos: diagnóstico, diseño, desarrollo y demonstración de impacto. El paso correspondiente al diagnóstico consiste en reunir en un seminario a los principales socios con el fin de establecer de manera colaborativa las barreras más importantes y ofrecer soluciones. En el paso de diseño, se elabora un plan específico para actuar conforme a las conclusiones del seminario de manera consensuada sobre los indicadores centrales. El paso de ejecución supone la aplicación del plan en las ubicaciones piloto, al tiempo que se fortalece la capacidad de los sistemas de salud en lo relativo a las pruebas de detección y el tratamiento de la enfermedad de Chagas. Finalmente, en el paso de demonstración de impacto se comparan los datos iniciales con los datos anuales posteriores a la ejecución para evaluar el progreso. En Colombia, este enfoque ha contribuido a simplificar los procedimientos de las pruebas de detección y brindar mayor acceso a la detección y el tratamiento de la enfermedad de Chagas en las comunidades piloto, si bien todavía hay retos que superar. El método de cuatro pasos es una de las diferentes vías para garantizar que los mejores productos de tratamiento y diagnóstico estén al alcance de las personas afectadas por las enfermedades tropicales desatendidas.

## RESUMO

A ampla maioria das pessoas com doença de Chagas não é diagnosticada nem é tratada. Para melhorar o acesso ao diagnóstico e ao tratamento desta doença, é necessário superar uma série de barreiras. Neste relato, é exposta uma abordagem colaborativa com o objetivo de vencer as barreiras e aumentar a disponibilidade do exame de detecção da doença de Chagas. As possíveis áreas de intervenção são selecionadas de acordo com a carga de doença, o apoio de defensores da causa e o compromisso das autoridades nacionais e locais. Uma abordagem em 4Ds (diagnóstico, desenho, desenvolvimento e demonstração de impacto) é implementada. No passo ‘’diagnóstico’’, um seminário com os principais atores é realizado para identificarem as maiores barreiras e proporem soluções de modo colaborativo. No passo ‘’desenho’’, um plano de ação é elaborado a partir das conclusões tiradas do seminário mediante o consenso sobre os indicadores básicos. No passo ‘’desenvolvimento’’, o plano é implementado em áreas-piloto e, em paralelo, a capacidade do sistema de saúde de realizar os exames de detecção e o tratamento da doença de Chagas é reforçada. Por último, no passo ‘’demonstração de impacto’’, os dados de referência são comparados aos dados anuais pós-implementação com o objetivo de avaliar o progresso. Na Colômbia, apesar de persistirem os desafios, esta abordagem contribuiu para simplificar o procedimento para o exame de detecção da doença de Chagas, aumentar o número de exames realizados e melhorar o acesso ao tratamento nas comunidades-piloto. A abordagem em 4Ds representa um dos vários cursos a serem seguidos para garantir que os melhores recursos diagnósticos e terapêuticos cheguem até as pessoas afetadas por doenças tropicais negligenciadas.

Over 6 million people worldwide are infected with *Trypanosoma cruzi* (1 – 3), the protozoan that causes Chagas disease (CD). *T. cruzi* is transmitted by triatomine insects, congenitally, through uncontrolled blood donations and organ transplants, and via consumption of food or drink contaminated by triatomines. The acute phase, though occasionally fatal, is often without recognizable symptoms. It is followed by a long, indeterminate period during which most people are unaware of their infection. However, heart failure and other life-threatening complications impact 30% – 40% of people with CD, usually decades later (4).

Since the 1990s, advances have been made in halting vector transmission and screening the blood and organ supply. However, progress in bringing treatment to affected people has been much slower, and CD remains one of the world’s most neglected diseases. Studies in Mexico, the United States, and Colombia indicate that fewer than 1% of people with CD have access to diagnosis and treatment (5 – 7). Diverse barriers perpetuate this neglect, including clinical challenges associated with diagnosing and treating CD (8), political and socioeconomic inequalities (9 – 12), and gaps in health care systems (5 – 7, 13). Moreover, the epidemiologic profile of CD has presented new challenges. Several outbreaks of orally transmitted CD, with a potentially severe acute phase, have occurred in Brazil, Colombia, and Venezuela (14, 15). Addressing the complex access challenges for CD requires long-term commitments from various stakeholders, including governments, researchers, patient associations, and non-profit organizations. Product development partnerships (PDPs), which have been instrumental in developing several new diagnostic and therapeutic products for neglected diseases in recent years, have a key role to play in ensuring patient access to testing and treatment. This paper describes a long-term approach developed by the Drugs for Neglected Diseases *initiative* (DND*i*) to improve access to treatment for people with CD. The goal here is to share lessons learned and to propose a straightforward methodology for addressing barriers. This approach is one among several potential public health solutions for CD.

## PRODUCT DEVELOPMENT PARTNERSHIPS AND CHAGAS DISEASE HEALTH CARE ACCESS

The PDP model, endorsed by the World Health Organization (WHO), has targeted the gap in neglected tropical disease (NTD) research. PDPs are not-for-profit organizations conjoining diverse actors in collaborative initiatives to develop drugs and diagnostics for underserved public health needs. The proportion of research projects devoted to neglected diseases from 2000 – 2011 (4%) was nearly 4-fold compared with 1975 – 1999, though a significant research gap remains (16). In 2012 – 2018, only 6 of 256 new pharmaceutical products targeted NTDs; all were repurposed drugs, new formulations, or biologicals. No new chemical entity for NTDs reached the market from 2000 – 2019 (17), when fexinidazole was approved for treatment of human African trypanosmiasis (18). In the case of CD, the only two effective drugs were developed prior to 1975.

PDPs are increasingly aware that developing and registering a new safe and effective treatment, while necessary, does not by itself ensure patient access (19). Access is defined as a scenario in which all patients can freely and promptly obtain appropriate health care without incurring undue hardship or financial burden. Several approaches describe dimensions involved in access to health care and related technologies as a basis for developing effective interventions, particularly in low- to middle-income countries (19 – 22).

How can PDPs impact health systems to ensure patients have access to the best available treatments, including recent products emerging from the research and development (R&D) pipeline? PDPs harness a broad range of expertise to transform health systems and enhance uptake of new products. They can assemble and disseminate data on health care system needs, raising awareness and potentially impacting country-level policies. However, PDPs may confront internal divisions between those who would focus strictly on product development and those who favor a more proactive role in making sure products reach end users (23). Moreover, there is an ongoing risk of imbalances in control and responsibility between local actors in endemic countries and PDPs, whose staff and leadership may be concentrated in the Northern Hemisphere (23, 24).

## DND*i* and access to Chagas disease treatment

Doctors without Borders/Médecins sans Frontières (MSF) created a working group to improve availability of treatment for neglected diseases and in 2003 seven founding members (https://www.dndi.org/about-dndi/founding-partners/) formed DNDi as an alternative R&D model. In the field, MSF had become acutely aware that market-driven R&D was failing to provide adequate therapeutic alternatives for diseases afflicting poor, underserved populations. Using an innovative model based on partnerships with a broad range of actors in academia, government, and the pharmaceutical industry, DND*i* has delivered eight new treatments for neglected conditions, most recently the first all-oral treatment for human African trypanosomiasis (25).

Since its founding, DND*i* has prioritized CD. DND*i* conducted the first major clinical trial in decades for a new chemical entity for CD, fosravuconazole (26). While this compound ultimately proved unsuccessful at sustaining parasite clearance, the trial did affirm the efficacy of benznidazole under strictly controlled conditions. Further, in a recently concluded DND*i*-supported trial, alternative regimens of benznidazole, including shorter courses, lower doses, and in combination with fosravuconazole, proved effective at clearing *T. cruzi* (27); another trial is evaluating fexinidazole (https://clinicaltrials.gov/ct2/show/NCT03587766).

Meanwhile, since the mid-2000s there has been a paradigm shift in CD treatment. Previously, treatment was typically offered only to children or acute cases. Recommendations did not support treating asymptomatic adults (most CD cases) because of a belief that advanced CD pathology was due principally to an overactive immune response (28). Since then, evidence has accumulated demonstrating that antiparasitic treatment with benznidazole and nifurtimox yields improved clinical outcomes in chronically infected adults, clears the parasite (a trigger of complications), and prevents future congenital transmission (26, 29 – 36). Several guidelines, including recent recommendations by the Pan American Health Organization (PAHO), support offering antiparasitic treatment to adults with chronic indeterminate CD (37 – 40). Importantly, antiparasitic treatment should be timely, i.e. prior to onset of cardiac complications, after which it appears to be less effective (41).

Many clinicians were slow to adapt, while others openly resisted the new paradigm (42). Resistance centers on doubts regarding the strength of evidence supporting treatment (stemming from observational studies rather than randomized clinical trials, and the lack of a reliable marker of cure), and concerns about adverse effects from antitrypanosomal drugs among older persons. MSF was an early advocate for scaling up treatment for adults. In collaboration with the Ministry of Health of Bolivia, MSF demonstrated the feasibility of providing treatment on a large scale to a vulnerable population of adults with CD in an endemic, rural area, with comparatively low rates of discontinuation (10.2%) secondary to adverse effects (43).

**FIGURE 1.**
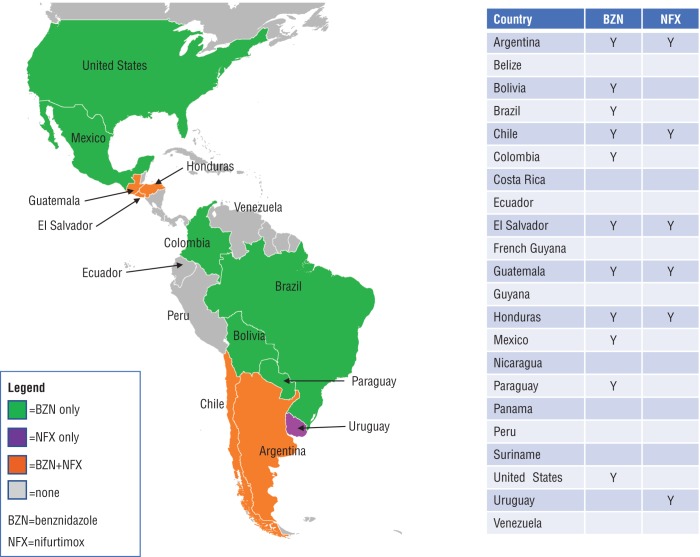
Registration of antitrypanosomal drugs in the Americas as of August 2019

***Source:*** Prepared by the authors based on information provided by InSud Pharma,® Buenos Aires, Argentina (benznidazole) and Bayer AG,® Leverkusen, Germany (nifurtimox).

The supply of antitrypanosomal drugs has improved in the last decade, with the emergence of two producers of benznidazole in the Americas and annual donations of nifurtimox from Bayer AG (Leverkusen, Germany) to WHO. Both drugs are now registered in several countries in the Americas (Figure 1), but are also available through the PAHO Strategic Fund. DND*i* has supported increased availability of benznidazole in health systems, and in 2011 launched and registered the first pediatric formulation in partnership with Laboratório Farmacêutico do Estado de Pernambuco (Recife, Brazil), a state-owned laboratory in Brazil (44).

Despite this progress, the number of CD patients receiving treatment remains low (45). This deficiency stems from various interrelated barriers, including the low priority given to CD by public health systems; the political marginalization and economic insecurity of the affected population (9, 11); low awareness among laypeople and physicians (13, 46, 47); diagnostic challenges (48); stigmatization and fear surrounding CD (12, 49); the lack of a test of cure (8); and the absence of treatment options in or near patients’ communities (10, 13). These barriers inhibit the demand for diagnostic and therapeutic products; in turn, low demand does not sustain production levels necessary for reaching millions of untreated individuals with CD.

While DND*i* has continued efforts to develop a new treatment, find an improved regimen of benznidazole, and identify effective biomarkers of treatment efficacy, in 2015 it launched an access initiative to explore and implement solutions to testing and treatment barriers. The access initiative has two primary goals: (i) improve patient access to existing tools, and (ii) pave the way for uptake of new drugs and diagnostics emerging from the R&D pipeline. An access strategy and methodology were developed in consultation with global experts involved in CD, and the first project was implemented in Colombia (50).

## STRATEGIC CONSIDERATIONS FOR ACCESS PROJECTS

This access strategy focuses on developing sustainable, collaborative solutions. Regional objectives for diagnosis and treatment of Chagas disease (Table 1) are supported through partnership with national and local health systems to address barriers (51 – 53). DND*i* opted not to implement a vertical strategy dependent on a large investment of external resources; instead, national and local health systems are upheld as the main protagonists. Project goals are driven by in-country partners. DND*i* acts as a catalyst, engaging in dialogue with national stakeholders so that CD receives a high priority on the research and public health agenda, while supporting collaboration among different sectors (e.g., researchers/academia, civil society groups, and public health officials). DND*i* provides technical support in identifying and addressing barriers, and in ensuring training and capacity building for health care personnel. Eventually, projects should become self-sustaining, without DND*i* involvement.

**TABLE 1.** Regional Chagas disease objectives that guided the Drugs for Neglected Diseases *initiative* projects for access to Chagas treatment

Source	Year	Objectives
Elimination of Mother-to-Child Transmission-Plus (51)	2017	- Ensure that ≥ 90% of infants and children with Chagas disease are treated and cured - Screen ≥ 90% of pregnant mothers - Treat ≥ 90% of seropositive mothers
The London Declaration on Neglected Tropical Diseases (52)	2012	- Sustain, expand, and extend drug access programs to ensure an adequate supply of drugs and interventions to control Chagas disease by 2020 - Provide technical support, tools, and resources to support NTD-endemic countries to evaluate and monitor NTD programs
Strategy and Plan of Action for Chagas Disease (53)	2010	- To reduce morbidity and mortality by improving access to health services for people infected with *T. cruzi*, both symptomatic and asymptomatic, and increase the coverage of diagnosis, quality medical care, and timely treatment of cases - To ensure diagnosis, medical care, and treatment of people infected with *T. cruzi* - 100% of countries to control congenital transmission - To perform technology research and innovation, with special emphasis on developing new drugs for etiological treatment

***Source:*** Prepared by the authors from the study data.

The decision to launch a project is not a unilateral imposition from DND*i*. Rather, it is dependent upon the interest and commitment of national and local governments, which are critical to achieving a successful outcome. There needs to be a local champion, such as an investigator, clinician, or public health official who embraces the project and works within the health system to introduce change (23). Other important considerations are the burden and distribution of the disease, existing capacity and infrastructure of the health system, health policy landscape, regulatory status of medications and diagnostic tools, local capacity for surveillance and operational research, and state of transmission control.

Table 2 lists elements that enhance the success of access projects. The strength and presence of these criteria vary considerably in different contexts. In Colombia, the Ministry of Health and Social Protection has actively supported the expansion of CD treatment. This benefits a population heavily impacted during the conflict, and fits well with Colombia’s peace process agenda. Colombia has further shown leadership in public health through an official policy of universal health coverage, elimination of onchocerciasis, and efforts to safeguard access to high-cost drugs.

**TABLE 2.** Key elements of the Drugs for Neglected Diseases *initiative* (DND*i*) Chagas treatment model as demonstrated by the Colombia pilot project

Key element	Colombia
Local capacity	The health system has the infrastructure in place to implement the access project
Political commitment	Addressing Chagas and other neglected diseases supports the government’s agenda to address such conditions
Scalable pilot projects	A new comprehensive roadmap for Chagas disease was initially implemented in four communities, and will be replicated in other communities
Partnership model	The project is led by the Ministry of Health and Social Protection, DND*i* provides technical support, other partners include the National Health Institute and state/local authorities
Sustainability	The comprehensive roadmap is supported by official policy and, once implemented, does not require DND*i*’s support
Local champions	Key persons within the health system have embraced the access project and advocated for its adoption and replication

***Source:*** Prepared by the authors from study data.

While several barriers may need to be addressed at the national level, such as updating or simplifying guidelines and ensuring an adequate supply of tests and medication, solutions are tested locally through small-scale pilot projects. This means that results are much slower to develop and the number of people tested and treated is initially small. The pilot projects are run by local health care personnel and driven by country-defined public health and political priorities. The function of the pilot projects is to demonstrate the feasibility of providing diagnosis and treatment within the existing health system in various contexts. Adjustments can be made based on lessons learned, and interventions can be fine-tuned for scaling up throughout the health system.

## The 4D access approach

Although local contexts and needs will shape project design, the DND*i* access methodology involves a “4D” process: diagnose, design, deliver, and demonstrate impact (Box 1).

BOX 1. **The 4-D process for implementing collaborative initiatives to increase access to diagnosis and treatment of Chagas disease**

**Table:** 

Step 1: Diagnose	• Gather information on the current state of prevention, diagnosis, and treatment in each country to establish baseline data. • Take inventory of the current organizations, programs, and resources devoted to Chagas disease. • Bring together multiple stakeholders from the health sector: government, civil society, academia, the private sector, international organizations, among others. • Collectively define the primary barriers to preventing, detecting, and treating Chagas disease.
Step 2: Design	• Develop customized access plans to overcome the country-level barriers identified in Step 1. • Align with other country-level and regional initiatives. • Collaboratively establish clear objectives and timelines. • Collect baseline data; ensure reliable data collection systems to measure impact. • Identify human resources and training needs.
Step 3: Deliver	• Implement the access plan. • Actively engage local stakeholders, encouraging local ownership. • Build capacity to foster sustainability; “train the trainers.” • Implement an information, education, and communication strategy.
Step 4: Demonstrate impact	• Collect data on the performance of the plan and monitor and evaluate results. • Retool and refine the plan as needed based on periodic evaluations. • Share evidence and lessons learned both locally and externally.

***Source:*** Prepared by the authors from study data.

### Diagnose.

The first step is to identify and map key stakeholders, gather epidemiologic and policy information, assess gaps and barriers, highlight opportunities/policy windows, and take stock of government engagement (e.g., commitment to United Nations Sustainable Development Goals or existence of social programs focused on the needs of populations impacted by CD). Political, environmental, and social factors affecting CD and access to treatment also need to be considered. Finally, quick wins or low-hanging fruit are identified to create short-term objectives with the potential to generate project momentum.

Initially, this information is gathered through consultation with local experts, ministries of health, academics, clinicians, and local WHO/PAHO officials in a situational assessment of the CD access landscape. An access seminar is held with key stakeholders, including government representatives, patients, researchers, civil society, public health officials, local WHO/PAHO representatives, and nongovernmental organizations. Its purpose is to reach a consensus on barriers affecting CD diagnosis and treatment and corresponding solutions. Intervention target-areas are identified, and ultimately selected with the agreement and involvement of local stakeholders. It is critical to ensure buy-in from all key decisionmakers to safeguard the project’s progress and sustainability.

### Design.

This step aims to design an actionable plan, shaped by the needs and priorities previously identified, in conjunction with the Ministry of Health and/or in-country partners. The plan should be comprehensive but pragmatic, incorporating evidence-based interventions and clearly outlining roles and responsibilities. It should use existing indicators based on national and international goals, including WHO and PAHO targets, adapted to local contexts. Other indicators and expected results should be agreed upon by all partners. What constitutes success may vary in different contexts. In Colombia, success was defined as creating and widely implementing a new, simplified patient roadmap (50).

How to measure impact needs to be addressed during this step; this may require actions to strengthen in-country surveillance and/or research capabilities. Clear objectives and time frames should be established. Baseline data are gathered and a reliable system of data collection is created using the agreed-upon indicators and project objectives. This system should be simple and user-friendly to encourage the buy-in of local health care personnel and facilitate cross-coordination among different sites. Important indicators might include the number of people tested and treated, the length of time between screening positive and receiving confirmatory testing, and number of health care personnel in each community trained in CD diagnosis and treatment. HR needs are identified, and a training framework is created to raise awareness in the health sector and to ensure good quality of care. Information, education, and communication (IEC) activities are created in this step, and should target both the population at risk and health care personnel.

### Deliver.

In this step, the pilot project is implemented and the IEC plan is launched, raising awareness among the at-risk population and increasing demand for diagnosis and treatment. Capacity building is an ongoing activity, but as the project matures, this responsibility shifts to local personnel, who become trainers of trainers. Data is systematically collected and periodically analyzed to assess project impacts and results and to address gaps or shortcomings. Policies, guidelines, and protocols may need to be modified (or new ones implemented) to create the conditions for the pilot to operate successfully. In Colombia, a simplified patient roadmap and diagnostic process were rolled out with the official validation of the Ministry of Health and Social Protection. Often, clinical guidelines will need to be updated to reflect the latest CD knowledge or to remove barriers; for instance, etiological treatment of patients with chronic, indeterminate CD could be shifted from the specialty level to primary health care. Threats to the drug or diagnostic supply can pose a major challenge during this step. It may be necessary to address regulatory and bureaucratic barriers in the supply chain or commit specific stock to the project.

### Demonstrate impact.

Monitoring and evaluation are critical in all phases of the project, and involve operational research to compare key indicators at baseline and after implementation. If required, DND*i* and/or partners provide initial operational research support and guidance. Local capacity for collecting and analyzing data is strengthened to the point of self-sufficiency. Results, experiences, and lessons learned are documented and widely disseminated. If gaps are identified, stakeholders may need to reconvene to agree on appropriate corrective actions. Evidence needs to be gathered and shared in a way that facilitates timely consumption by national policymakers, WHO, PAHO, and other stakeholders. The experience of successful projects should be actively disseminated to demonstrate feasibility and to facilitate replication in similar contexts.

## 4D approach in Colombia

In 2015, a seminar on barriers to treatment of Chagas disease was held in Bogotá with participants from academia, civil society, government, and health care (50). Participants discussed diagnosis and treatment barriers from the perspectives of patients, providers, and insurers. The patient perspective was a critical component. Representatives from two patient associations from endemic areas and individual patients residing in Bogotá participated in the seminar. Because complexities in the diagnostic process were identified as a key barrier, an early project activity involved development and validation of a simplified diagnostic algorithm, led by the National Health Institute of Colombia. The patient-centered roadmap for CD, accompanied by an IEC strategy, was piloted and validated in four communities; a fifth community was added later. Since 2016, several training and capacity building workshops have taken place in the pilot communities as well as departmental capitals and Bogotá. Some physicians received training within the Bolivian Chagas Platform (54) from clinicians with extensive experience in treating CD. Baseline data was collected on several operational indicators and is being compared to the performance of the pilot (Table 3). After a year, in the first two communities where the pilot project was implemented, mean delays in the diagnostic process were reduced from 364 days to 17, and the number of people tested annually increased from 35 to 384 (55). More people also received antiparasitic treatment, but a significant gap remained between the number of positive test findings and the individuals initiating treatment.

**TABLE 3.** Core indicators of health system capacity for ensuring patient access to diagnosis and treatment of Chagas disease, Colombia pilot project

Measure	Indicator
Number of patients:	• total tested for *T. cruzi* • pregnant women tested for *T. cruzi* • with confirmed positive diagnosis • with discordant or inconclusive results requiring a third test • receiving consultation after positive diagnosis • eligible/ineligible for etiological treatment • initiating etiological treatment • discontinuing treatment due to side effects • referred for specialist care due to complications from advanced chronic CD • with annual follow-up visits
Days between:	• solicitation of testing and confirmed diagnosis • confirmed diagnosis and initiation of etiological treatment
Other	• facilities within the diagnostic network • health care personnel receiving specific training for Chagas through the pilot
Key patient variables	• gender • community of residence • insurance status (private or subsidized) • age

***Source:*** Prepared by the authors from study data.

## DISCUSSION

The DND*i* access model takes a long-term view and starts small-scale, leveraging local resources to gradually transform health systems from within. This 4D approach provides a simplified, straightforward methodology for launching and sustaining collaborative projects that address CD access issues. However, this approach can only work as a partnership in which governments and local stakeholders are invested and involved. The principle contribution that DND*i* makes is helping strengthen in-country capacity to manage, and eventually scale up, pilot projects by providing training, advice, and guidance. Another DNDi role is supporting epidemiologic surveillance and helping anticipate the demand for drugs and diagnostics through data collection in pilot communities.

The project in Colombia has improved access to testing and treatment in the pilot communities, but the number of people impacted remains small. Scaling up the patient-centered roadmap on a national level, the goal of the project, could eventually create a significant impact. However, ongoing challenges remain: ensuring that patients with a confirmed CD diagnosis promptly initiate treatment, assuaging providers’ concerns regarding adverse effects, and raising awareness of available and effective treatment among at-risk communities. Addressing these challenges requires continued dialogue and collaboration with local and national authorities, and sustained, long-term IEC, training, and capacity building activities.

The implementation of the pilot project in Colombia has provided important insights. While providing CD testing and treatment within the established health system enhances the long-term sustainability and cost effectiveness of the project, it may require a lengthier implementation period. Ideally, IEC activities should be implemented at an early phase, and IEC materials should be tested with patients and community members. Macro-level indicators, such as the supply and distribution of trypanocides and diagnostic products, should be continually monitored along with specific indicators of patient care at the local level. Training and capacity building need to be ongoing activities because health care personnel in rural areas may rotate frequently.

DND*i* is also involved in activities to improve access to CD treatment in the United States, and has begun 4D methodology projects in Guatemala and Brazil; each of these contexts has particular needs and challenges. Other collaborative approaches have successfully increased access in different contexts. The experience of MSF in rural Bolivia and elsewhere showed it was possible to provide antitrypanosomal treatment and monitor side effects in rural, disadvantaged communities. Klein and colleagues (56) described access barriers in primary health care facilities in Argentina, as well as a pilot program to decentralize CD treatment. The Mundo Sano Foundation, in conjunction with the local health department, launched another pilot program focused on strengthening treatment at the primary health care level in La Plata, Argentina (57). The Bolivian Chagas Platform, a collaborative initiative between the Barcelona Institute for Global Health (Barcelona, Spain) and the *Fundación Ciencia y Estudios Aplicados para el Desarrollo en Salud y Medio Ambiente* (Cochabamba, Bolivia), developed a model of care for CD in conjunction with academic institutions and the Ministry of Health of Bolivia. Based on four pillars (providing health care, building research capacity and clinical expertise, training health professionals, and community education), the Bolivian Chagas Platform has treated over 8 500 people with CD and trained over 1 600 health professionals (54).

### Conclusions

PDPs are well positioned to foster synergies among the multiple stakeholders that play a vital role in tackling barriers to the diagnosis and treatment of neglected diseases. Access issues are complex and multidimensional; solutions must harness the latest scientific knowledge as well as transform health systems and mobilize civil society. The DND*i* access strategy complements its R&D efforts and provides one potential pathway to creating a favorable environment to ensure drugs and diagnostics reach patients. Other successful models exist, and new approaches should be implemented and tested under real conditions in diverse contexts, so that ultimately the fruits of scientific innovation are equally accessible to all.

### Author contributions.

CB conceived of the original idea and prepared the manuscript. CJF reviewed the literature and prepared the manuscript and tables. RH collected data and prepared the tables. MC collected data and prepared the figure. AM collected data and prepared the tables. All authors reviewed the manuscript and approved the final version.

### Acknowledgements.

We would like to thank our partners in Colombia and elsewhere for tirelessly working to make treatment for Chagas disease more accessible for affected people. Many thanks to Mauricio Vera of the Ministry of Health and Social Protection of Colombia, to Carolina Florez and Andrés Caicedo of the National Health Institute, and to the Secretaries of Health and numerous others Arauca, Boyacá, Casanare, and Santander, whose dedication made the pilot project in Colombia possible. Special thanks also to Carlos Valencia for his early work on the access project, and to Mae Shieh and Sergio Sosa-Estani of DND*i* for helpful suggestions on the 4D methodology.

### Funding.

DND*i* is grateful to its donors, public and private, who have provided funding to DND*i* since its inception in 2003. A full list of DND*i* donors can be found at http://www.dndi.org/donors/donors.

The funders had no role in the study design, data collection or analysis, decision to publish, or preparation of the manuscript.

### Disclaimer.

Authors hold sole responsibility for the views expressed in the manuscript, which may not necessarily reflect the opinion or policy of the *RPSP/PAJPH* and/or PAHO.
